# Exploring the information transmission properties of noise-induced dynamics: application to glioma differentiation

**DOI:** 10.1186/s12859-019-2970-7

**Published:** 2019-07-04

**Authors:** Aditya Sai, Nan Kong

**Affiliations:** Weldon School of Biomedical Engineering, Purdue University, 206 S Martin Jischke Drive, West Lafayette, 47907 IN USA

**Keywords:** Information theory, Mutual information, Channel capacity, Stochastic modeling, Chemical langevin equation, Glioma differentiation, *k*-nearest neighbors, *k*-means clustering

## Abstract

**Background:**

Cells operate in an uncertain environment, where critical cell decisions must be enacted in the presence of biochemical noise. Information theory can measure the extent to which such noise perturbs normal cellular function, in which cells must perceive environmental cues and relay signals accurately to make timely and informed decisions. Using multivariate response data can greatly improve estimates of the latent information content underlying important cell fates, like differentiation.

**Results:**

We undertake an information theoretic analysis of two stochastic models concerning glioma differentiation therapy, an alternative cancer treatment modality whose underlying intracellular mechanisms remain poorly understood. Discernible changes in response dynamics, as captured by summary measures, were observed at low noise levels. Mitigating certain feedback mechanisms present in the signaling network improved information transmission overall, as did targeted subsampling and clustering of response dynamics.

**Conclusion:**

Computing the channel capacity of noisy signaling pathways present great probative value in uncovering the prevalent trends in noise-induced dynamics. Areas of high dynamical variation can provide concise snapshots of informative system behavior that may otherwise be overlooked. Through this approach, we can examine the delicate interplay between noise and information, from signal to response, through the observed behavior of relevant system components.

**Electronic supplementary material:**

The online version of this article (10.1186/s12859-019-2970-7) contains supplementary material, which is available to authorized users.

## Background

Cells engage in dynamic interactions with their environment, from which they receive and transmit information in the form of biochemical signals, in order to sense and respond physiologically to changing conditions. However, the normal propagation and processing of these signals can be hindered by the presence of biochemical noise, which can be decomposed into cell-to-cell variability (extrinsic noise) and stochastic intracellular fluctuations (intrinsic noise) [1, 2]. In spite of this noise, robust and reliable information transmission is critical for directing the cellular decisions necessary for environmental adaptation and survival [1, 3]. Therefore, it is beneficial to quantify the accuracy and efficiency by which a given signaling pathway relays information from the external environment into the cell interior.

Information theory was developed in the late forties by Shannon to study information transmission across man-made communication channels [4]. When applied to biochemical signaling pathways, it can be used to determine the number of physiologically distinct states necessary to fully capture a distribution of responses, often sampled from a population of genetically identical cells exposed to the same stimulus. While conventional statistical measures, such as the mean and variance, may capture the magnitude of noise, they do not reflect the degree to which noise prevents discrimination of different stimuli or the accuracy of information processing at the single cell level [3]. On the other hand, information theoretic measures require no mechanistic knowledge [3], and have been found to be less sensitive to network perturbations than the mean signal intensity, at the population level [5]. Understanding biological information processing at the molecular and cellular level requires the ability to evaluate the efficiency of signal transduction processes, a task information theory is uniquely suited for [1].

Mutual information specifies the statistical dependence between two random variables by measuring how much information is preserved from input (signal) to output (response). In the context of cell populations, mutual information can indicate the number of different signals the cell response data can adequately resolve. Besides describing the quality of information transfer within signaling networks, mutual information has also been used to reverse-engineer signaling networks [6], and design optimal experiments for parameter inference [7]. Since the mutual information of a pathway is rarely known in vivo, it is customary to compute the maximum mutual information value over all possible signal distributions, known as the channel (or information transmission) capacity. The channel capacity serves as a fundamental feature of the signaling channel between signal and response. While it is formulated as an upper bound on the amount of information transmitted through a channel, the channel capacity is practically considered a lower bound on information content due to the presence of noise [3]. The mutual information and channel capacity of a signaling pathway can be useful in quantifying the information content in complex processes, such as cancer, where the flow of normal biological information is disrupted [8, 9].

In this study, we present an information theoretic approach to evaluating the noise-induced dynamics of two stochastic models of glioma differentiation, the additive noise (AN) model and the chemical Langevin equation (CLE) model [10]. By considering multiple input and noise levels, we compute the channel capacities of the glioma differentiation pathway using both summary descriptors and multivariate vectors representing response data. Weakening ultrasensitive, positive feedback mechanisms of certain upstream components actually improves signal fidelity. We additionally explore strategies to maximize information transmission by prioritizing different aspects of the differentiation response when computing the channel capacity. We increased the channel capacity of the CLE model by selecting time points with maximum variance for inclusion in the multidimensional response vector. Clustering response dynamics based on their relative activation to each signal reveals distinct classes of information transfer. Through this case study, we demonstrate applicability of information theoretic analysis to similar models of signaling pathways using stochastic differential equations (SDEs). While there have been previous applications of information theory to stochastic models [7, 11], we present a comprehensive framework with which to apply information theoretic measures to biologically relevant systems and explore tuning algorithm parameters to maximize channel capacity.

## Methods

### Glioma differentiation model

**Fig. 1:**
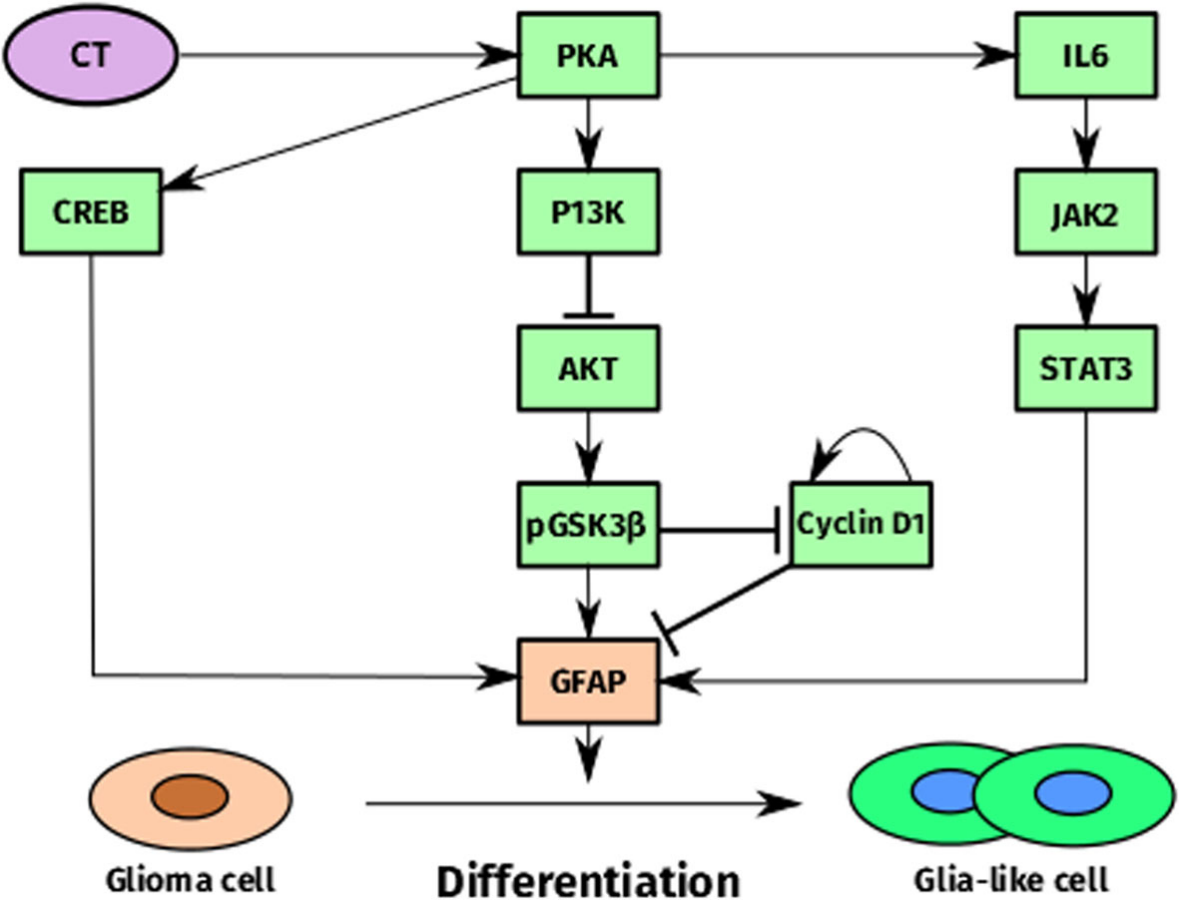
Glioma differentiation signaling network. Cholera toxin (*purple*) acts as a principal input to the system, inducing glioma cell differentiation via multiple pathways, the PKA/CREB [13], P13K/AKT/pGSK3 *β*/cyclin D1 [14], and IL6/JAK2/STAT3 [15] pathways. GFAP (*pink*) serves as the differentiation marker, measuring the extent to which glioma cells differentiate into normal glia-like cells

Glioma differentiation therapy is an alternative to surgery, radiation, and chemotherapy in cancer treatment [12]. Cholera toxin (CT) was found to induce glioma cell differentiation, producing non-cancerous glia-like cells [12]. A deterministic model initially incorporated multiple interacting pathways [13–15] involved with CT-induced differentiation, in order to clarify the underlying molecular mechanisms [16]. This integrated pathway is shown in Fig. 1. An irreversible bifurcation switch controlling the phenotypic transition from proliferation to differentiation was discovered, attributed to the ultrasensitive response of cyclin D1 to CT treatment. Cyclin D1 dynamics were also found to be correlated with those of gilial fibrillary acidic protein (GFAP), a cell differentiation marker. The initial model accounted for these observations by integrating a positive feedback loop of cyclin D1, which when downregulated by cyclin D1 translocation and degradation, induces higher GFAP levels and differentiation.

The glioma differentiation models are Itô stochastic differential equation-based models, each consisting of 10 model states, 41 model parameters, and 1-2 noise parameters. They are described in Additional file 1. The AN model introduced in [10] accounted for stochastic interference in the signaling pathway by employing additive noise in the form of Brownian motion, resulting in SDEs. Higher noise intensities reduced the differentiation potential (defined as the percentage of the cell population to reach GFAP values of 0.8), induced heterogeneity, and enhanced drug resistance to differentiation-inducing drugs like CT. The model reaffirmed the ultrasensitivity of cyclin D1 to CT by fitting highly specific Hill coefficients in its response to CT induction. Inhibiting cyclin D1 feedback was found to decrease the heterogeneous response and improve anti-cancer drug efficacy. Noise-mitigating interventions were recommended as an effective solution to promote glioma differentiation. However, this model contains constant noise terms, which may not fully resemble stochastic signal transduction processes, as was pointed out [10].

The CLE model, also proposed in [10], included multiplicative noise terms for both extrinsic and intrinsic noise sources that relied on protein concentrations. Based on the white-noise version of the chemical Langevin equation, the model predicted reductions in differentiation potential when at least one noise source was increased above its baseline level. We also explore the information transmission of a modified version of the CLE model that inhibits positive feedback of cyclin D1, which we term CLE-. When compared to the CLE model, the CLE- model enhanced differentiation outcomes for the population by increasing GFAP activity and reducing heterogeneity, implicating the ultrasensitivity of cyclin D1 to CT for therapeutic inefficacy [10].

In this work, we produced an ensemble of continuous GFAP response data for analysis, corresponding to a population of 500 glioma cells, simulated with each of the three models. GFAP dynamics were simulated in response to 16 specific signals for 48 hours. Each signal was composed of a distinct CT dose and noise level. We considered 4 discrete CT doses of 0, 5, 7.5, and 10 ng/ml, previously explored in [10, 16]. These doses were applied continuously from the start of the simulation. For the AN model, noise intensities of 0.1, 1, 5, and 10% were applied. For the CLE and CLE- models, we specified values for both the intrinsic and extrinsic noise (Table 1). Mutual information, in this context, characterizes how accurately time-varying trajectories of downstream proteins, like GFAP, can discern differences between concentrations of upstream ligands, like CT and noise. The entire algorithm and models were coded and implemented in MATLAB.

**Table 1:** Noise Levels for CLE and CLE- Models. Noise levels indicate standard deviations of intrinsic and extrinic noise

Noise Level	Intrinsic Noise	Extrinsic Noise
LL	0.001	0.001
HL	0.1	0.001
LH	0.001	0.1
HH	0.1	0.1

### Multivariate channel capacity algorithm

In order to quantify the information transmission capabilities of the glioma differentiation pathway, as interpreted by our target models, we implemented a channel capacity algorithm, proposed by [17], which maximizes the mutual information between a vector (response dynamics) and a scalar (signal values). The response vector contains single cell responses at multiple time points. Multivariate formulations of the channel capacity were able to reduce information loss due to extrinsic noise substantially by incorporating more information from multiple time points, exploiting the dependency in response dynamics [17]. This additional information sufficiently resolved overlapping response distributions in higher dimensions arising from different signals, reducing the effects of extrinsic variability.

The algorithm first estimates the conditional probability density for each cell’s response, characterized by a multidimensional vector, using *k*-nearest neighbors density estimation. To form this response vector, continuous response data are subsampled uniformly around the middle time point to the desired resolution. Then, the entropy of the response, and the conditional entropy of the response given the signal, can be determined. The difference in these two terms gives the mutual information, which can then be maximized over all possible probability distributions of the signal, using the MATLAB optimization function *fmincon*, to obtain the channel capacity.

Channel capacities of scalar descriptors characterizing each cell’s individual GFAP trajectory were also calculated for comparison to those computed from the trajectories themselves. We considered three different scalar descriptors for this work:

1. the maximum GFAP level (max response),
2. the ratio of the maximum GFAP level to the initial GFAP level (max fold change), and
3. the area under the curve (AUC), computed using the MATLAB integration function *trapz*.

Summary descriptors contained lower information transmission capacities compared to their multivariate counterpart [17–19]. In addition, we explored normalizing each cell’s time course by the initial GFAP level, a fold transformation that improved channel capacities in other signaling pathways [18].

As in previous implementations of the channel capacity algorithm [17, 18], we had to first determine adequate values for both *k*, the number of nearest neighbors to consider for computing the conditional probability density of the response, and *d*, the dimension of the multivariate response vector. The search for these values is shown in Additional file 1: Figures S5-8. Tuning the value of *k* did not substantially alter channel capacity values, so we set *k*=5 in accordance with a previous study [18]. Channel capacities for different values of *d* converged to a maximum when dynamics from 5-6 time points were sampled for the response vector. As a result, we set *d*=6.

## Results

### Changes in response dynamics are most distinguishable at low noise levels

**Fig. 2:**
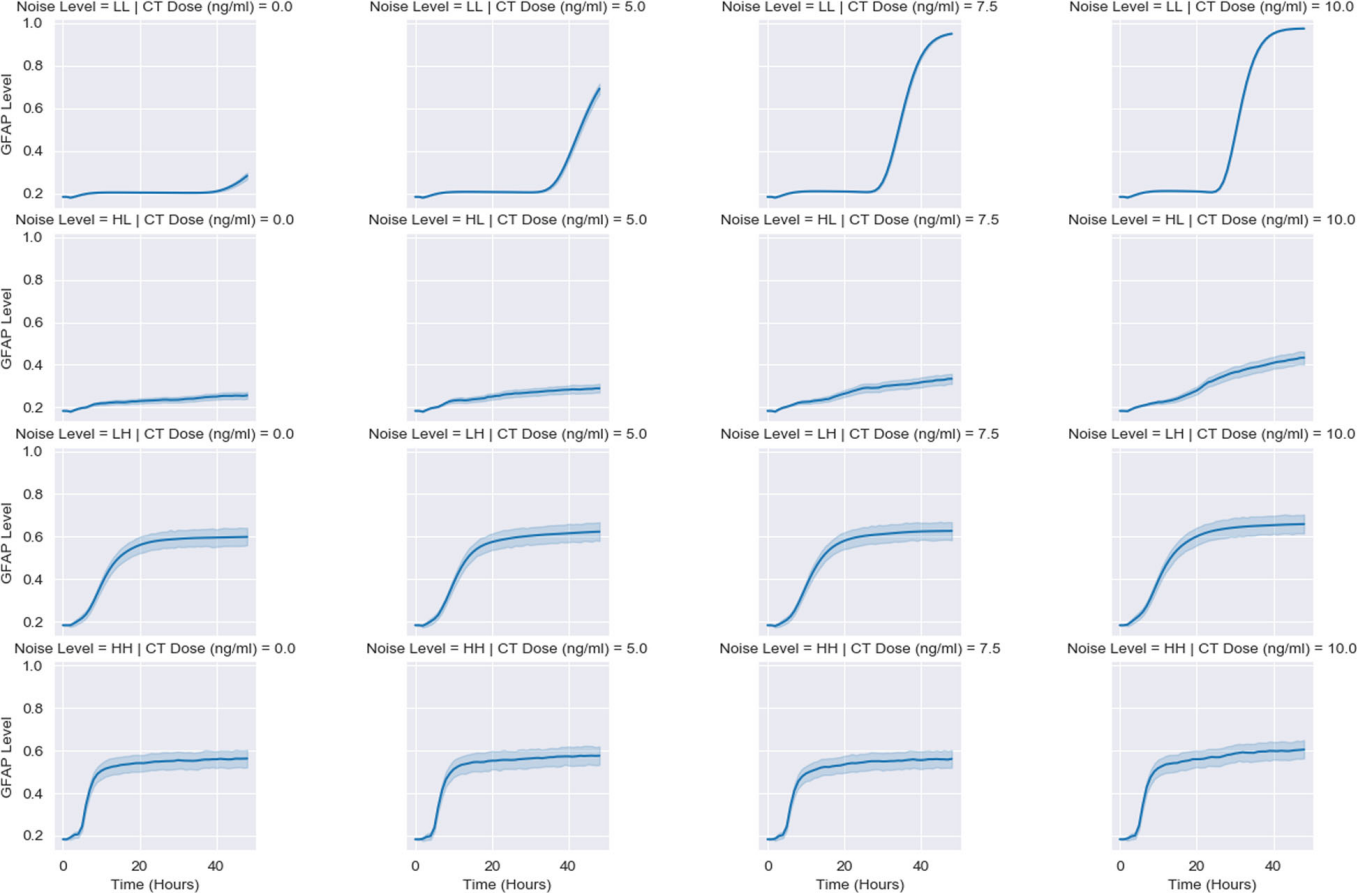
CLE response dynamics. Time courses of GFAP level are shown, corresponding to 500 simulated cells exposed to different signals composed of CT dose and noise (intrinsic & extrinsic noise). Dark blue lines represent average GFAP level, with shaded areas indicating 95% confidence intervals. Noise levels are defined in Table 1

**Fig. 3:**
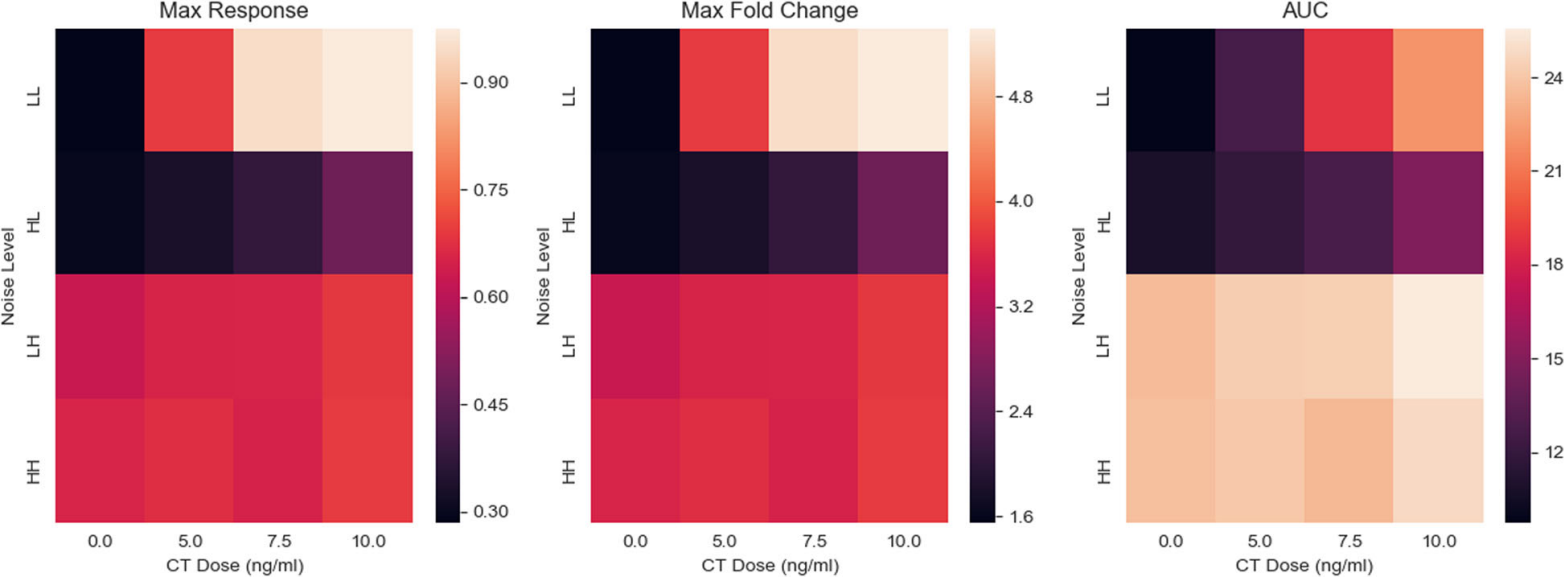
Heatmaps for summary descriptors of CLE model. Average maximum response (*left*), maximum fold change (*center*), and area under the curve (*right*) values were calculated for the simulated cell population exposed to each signal. Noise levels are defined in Table 1

To obtain the response data, we simulate the dynamics of each target model for different signal values, in order to observe how dynamics vary across CT doses for a given noise setting. Figure 2 features the response dynamics for the CLE model, arranged by noise level and CT dose. At a low intrinsic noise, low extrinsic noise setting (LL), almost all cells have become differentiated as CT dose gradually increases. Once the intrinsic noise is increased, a dramatic decrease in the GFAP response is observed, with a rapid decline in the differentiation potential. The final two rows of Fig. 2 show how extrinsic noise defines the response. These trajectories are seemingly invariant to the presence of intrinsic noise, reacting more to the external variability in seemingly identical cells. Increased intrinsic noise only serves to quicken the ascent to a plateauing of GFAP levels, but otherwise, the trajectories appear identical. Extrinsic noise predominates intrinsic noise when both are raised to higher levels. Summary descriptors applied to the CLE model also confirm these trends (Fig. 3). The mean max response, max fold change, and AUC descriptors show the greatest sensitivity to CT dose at the LL noise setting. Low levels of intrinsic and extrinsic noise discriminate between competing CT doses the best. However, this dose discrimination ability abates as the noise levels increase. For the CLE model, increased noise diminishes sensitivity to CT dose.

Noise has a more pronounced effect on the AN model (Additional file 1: Figure S1). A spectrum of GFAP activity was found, spanning from no GFAP activity to complete differentiation. Higher noise intensities disordered the GFAP distributions at earlier time points, resulting in divergent segments of the population with both increased and decreased activity. When cyclin D1 feedback was strongly inhibited, CLE- model dynamics show increased differentiation efficiency regardless of noise level and CT dose (Additional file 1: Figure S3). Maintaining robustness in the face of intra- and extracellular perturbations is accomplished by elucidating the pathway from CT to GFAP, resulting in increased GFAP activity. Both the AN (Additional file 1: Figure S2) and CLE- (Additional file 1: Figure S4) models show broader ranges of values when summary descriptors are applied. The CLE- model had higher average values for these descriptors compared to the CLE model, whereas the AN model expressed a broader range of descriptor values.

### Differences in static and vector channel capacities can be attributed to model structure

**Fig. 4:**
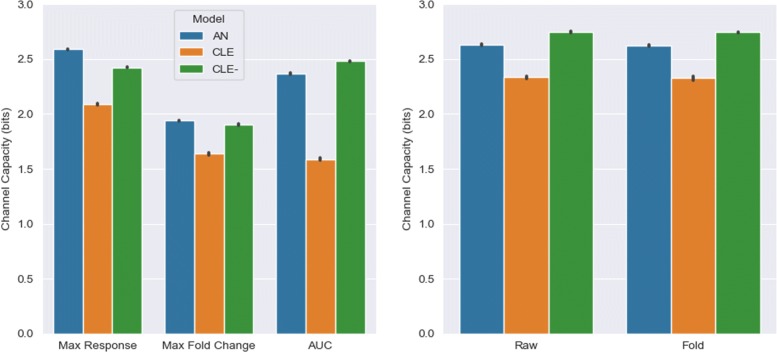
Information transmission for static and dynamic response data. Channel capacity values were calculated for static summary descriptors (*left*) and multivariate vectors representing GFAP dynamics (*right*), for all target models. Vector channel capacity values were calculated for both original (Raw) dataset, and fold-transformed (Fold) dataset. Results represent mean and standard deviation of 10 replications

We then calculated channel capacities when the summary descriptors were used to describe model dynamics, shown in Fig. 4. The channel capacities for the three descriptors computed across the three models estimate approximately between 1.5 to 2.5 bits of information flow from signal to response, meaning approximately 3-6 composite signals could be derived from these descriptors. The maximum response value transmitted more information on average for the AN (2.59 bits) and CLE (2.09 bits) models, while the AUC carried the most information for the CLE- model (2.48 bits). For the max fold change, there was less than 2 bits of information available for resolving signals, possibly because these values showed fairly small variation across signals. For each model, the channel capacities for the max fold change were significantly lower than those for max response and AUC (*p*<10^−4^, t-test). Furthermore, for each descriptor, the CLE model contained a lower channel capacity value compared to the other two models (*p*<10^−4^, t-test).

Multivariate calculations of the channel capacity using both the original and fold-transformed response dynamics demonstrated an increase in information transmission, corroborating prior studies [17–19]. The AN (2.63 bits), CLE (2.33 bits), and CLE- (2.75 bits) models showed visible improvements in average channel capacity once more time points were incorporated. For each model, the vector channel capacities were significantly higher than the static values (*p*<10^−4^, t-test). Again, the CLE- and AN models outperformed the CLE model in transferring more signal information onto the response. Weakening a critical positive feedback in the glioma differentiation model enhanced differentiation outcomes for the CLE- model, thereby improving information transmission. Likewise, the AN model induced sufficiently heterogeneous dynamics at each distinct noise level, enabling higher levels of activation and channel capacity. Finally, Fig. 4 proves that, unlike [18], no significant differences were observed by fold-transforming the response dynamics for these models.

### Asymmetric response vector sampling improves information transmission

Previous computations of the multivariate dynamic channel capacity relied on sampling the response vector symmetrically around the middle time point [17, 18]. Increases in the sampling rate improved information transmission, and that the tradeoff between low sampling rates (sampling dynamics that have already attenuated) and high sampling rates (redundant information) could reveal an optimal rate for maximizing channel capacity [19]. However, instead of focusing on periodic, uniform sampling techniques, we sought to determine whether asymmetric sampling focused on dynamical regions with maximum variation could improve the channel capacity. Two asymmetric sampling techniques were devised for comparison:

1. balanced sampling, in which dynamics from the time point with maximum variance in each of *d* equally sized subintervals were sampled, and
2. greedy sampling, in which dynamics from the *d* time points with maximum variance from the entire time interval were sampled.

**Fig. 5:**
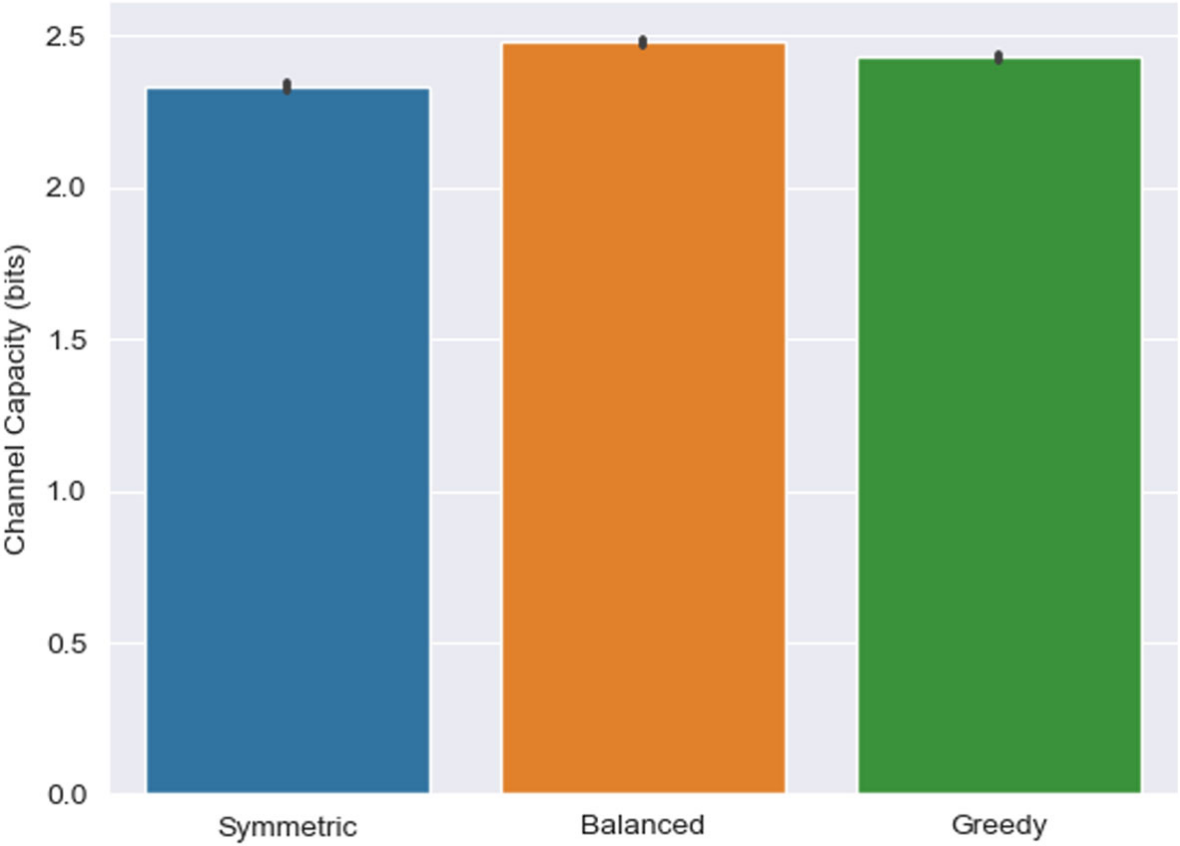
Information transmission for different multivariate vector sampling strategies with the CLE model. The default symmetric sampling method was compared against balanced (uniform sampling of maximum dynamical variation across time course) and greedy (non-uniform sampling) asymmetric methods. Results represent mean and standard deviation of 10 replications

Figure 5 highlights the results from comparing the default symmetric sampling strategy with our asymmetric variants for the CLE model. Gains of 0.15 and 0.09 bits were reported for the balanced and greedy sampling methods, respectively. Both variants displayed a significant increase in maximum information transmission compared to the default (*p*<10^−4^, t-test). Sampling dynamics that display maximum variation appears to add more value in terms of relaying information from signal to response. An increase in noise produces more variability in the response, further enabling differences in signals to be teased out from the response data as compared to a uniform sampling regime. Balanced sampling slightly edges out greedy sampling, implying that equal consideration for the variability across the entire time interval provides a greater channel capacity.

### Removal or partitioning of response data reveals subpopulations with distinct channel capacities

Removal of cell subpopulations nonresponsive to input stimulation were found to improve the channel capacity [18]. Likewise, we removed cells from the CLE model that failed to differentiate to determine their effect on channel capacity. That is, all cells whose GFAP levels failed to reach the threshold value of 0.8 by the end of the time interval were removed from the channel capacity calculations. However, the channel capacity of the terminally differentiated subpopulation failed to match that of the entire population, barely surpassing 2 bits (Fig. 7).

**Fig. 6:**
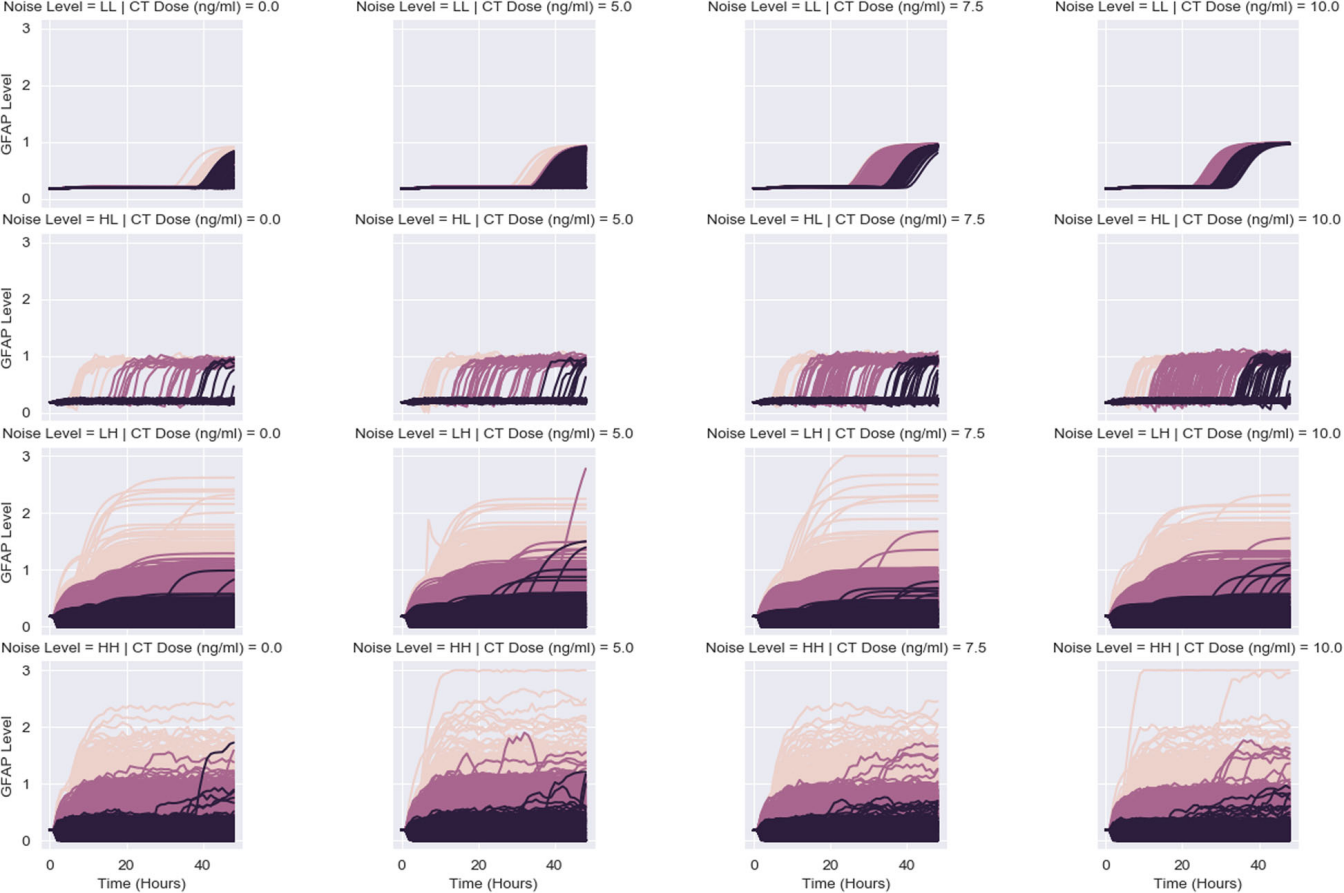
CLE response dynamics arranged by cluster. Time courses of GFAP level are shown, corresponding to 500 simulated cells exposed to different signals composed of CT dose and noise (intrinsic & extrinsic noise). Dynamics are colored by cluster membership

One of the issues in identifying a fully differentiated subpopulation is that stochastic modeling may prevent classification of a cell as fully differentiated due to the stochastic fluctuations in the GFAP level of a single cell. Cases of false positives (cells having little to no GFAP activity until the end of the time interval) and false negatives (cells having high GFAP activity that decline below the threshold at the last minute) may complicate identification of the differentiated subpopulation and calculation of its information transmission properties. Therefore, we resorted to clustering cells based on their response values across the entire time course, rather than at a single time point. We clustered the response trajectories corresponding to each signal into three clusters using *k*-means clustering. In order of descending average GFAP level, we labeled clusters C1, C2, and C3. Figure 6 illustrates the resulting clusters and their trajectories. When both the extrinsic and intrinsic noise are low, the clusters were not well separated. However, higher noise levels resolved the clusters fairly well.

**Fig. 7:**
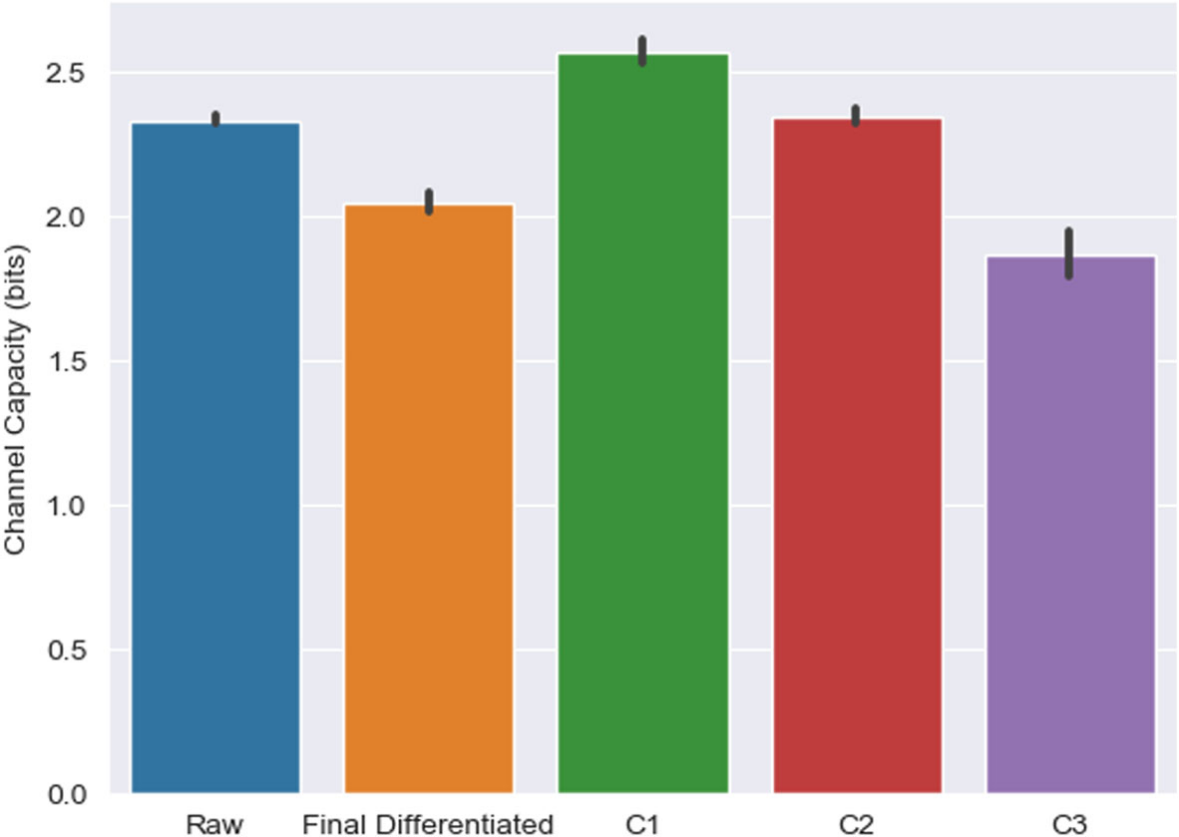
Information transmission for original and modified response data with CLE model. Channel capacity values were calculated for original (Raw) dataset, dataset with cells that reached differentiation threshold at end of simulation (Final Differentiated), and datasets representing distinct dynamical clusters (C1, C2, and C3). Results represent mean and standard deviation of 10 replications

Separation of the original dataset also resulted in separation of the channel capacities, into three distinct values. C1, C2, and C3 possessed average information transmission capacity values of 2.57, 2.35, and 1.87 bits, respectively. Figure 7 shows that C1 and C3 were found to have significantly different channel capacities compared to the original dataset (*p*<10^−4^, t-test). C1 represents the subpopulation with the highest GFAP levels and most likely to be fully differentiated, while C3 contains cells likely to be nonresponsive to signal stimulation. Isolating divergent cell dynamics facilitates increased knowledge of the signal to be passed along to the response as the C1 cluster captures a unique set of cells based on their entire response trajectory. On the other hand, the channel capacity of C2 was found to be statistically insignificant compared to that of the original dataset (*p*>0.05, t-test), implying that while the other two clusters represent the extremes of the differentiation spectrum, C2 is more representative of the entire dataset at large.

## Discussion

Noise distorts normal cell function and communication, confounding reliable signal resolution given response data. Nevertheless, most signaling pathways have evolved structurally and functionally to protect against noise to ensure information is relayed accurately from the extracellular environment to the cell nucleus. Furthermore, there is even an evolutionary justification for the presence of noise to expand the range of phenotypes in fluctuating environments [20]. However, it is important to understand the extent to which the underlying information may be compromised by noise and to determine whether a cell can communicate accurately in an unpredictable environment. Information theory provides a simple and straightforward approach to quantify the amount of information transmitted through these signaling pathways. Any complex system can be reduced to a black box communications channel for a rigorous evaluation of how information is encoded, transmitted, and decoded. Our work presents a viable information theoretic framework to analyze signaling network models, and can likewise be applied to similar systems where noise plays an active role in influencing the dynamics of key system components.

By treating noise as an element of a biochemical signal, we have normalized noise as a biological condition. Our in silico approach explicitly considered different noise conditions in formulating these signals, allowing for a comprehensive analysis of minimally to heavily perturbed response data. There are scenarios where the presence of noise may propagate through to the response dataset implicitly, so such dynamics will always have to be accounted for. The variation in a signal will always impact the reliability of its transmission more than its intensity [5]. From analysis of the response dynamics of the CLE model, the combination of both intrinsic and extrinsic noise is obviously non-additive. Extrinsic noise obfuscates the interpretation of channel capacity as all dynamics depend on model parameters perturbed by extrinsic noise [11]. Undoubtedly, increasing the dimension of the multivariate vector when computing the channel capacity alleviates the effects of intrinsic, and to a larger extent, extrinsic noise [17, 19].

Considering all of our target models, the CLE model transmitted the least information from signal to response on average, regardless of what information was used to compute the channel capacity. The CLE- model weakened negative regulation of the differentiation marker GFAP, relieving a *de facto* information bottleneck [21]. It is most likely the case that the CLE model serves as a negative feedback variant of the CLE- model. Negative feedback was found to initially increase dynamical variation, and channel capacity, but display the opposite patterns over longer periods of time [21]. By inhibiting positive feedback of cyclin D1, higher degradation rates of cyclin D1 (a consequence of the CLE- model) promote greater GFAP activity and less uncertain GFAP distributions. Our results underscore the importance of cyclin D1, an upstream regulator of GFAP, in characterizing the differentiation response and information flow in this signaling network. Similarly, the AN model, with its generic treatment of noise, also has a higher level of activation and information capture. However, gains in information exhibited by this model can be easily attributed to the disorganization introduced by an artificial noise source that is harder to actualize in a real-world setting. Furthermore, its predictions were suspect when inhibition of cyclin D1 feedback was implemented [10].

Sampling dynamics irregularly for inclusion in the response vector improved information transfer modestly. In particular, we found accounting for dynamical variation evenly across time led to more information being transferred. Our findings as it relates to asymmetric sampling agree well with previous results that suggest sampling dynamics in regions where they are most receptive to the signal will increase the channel capacity [19]. In the future, we may consider feature selection or dimensionality reduction techniques that identify optimal time points for better discrimination of response dynamics arising from different signals.

We segregated nonresponsive (potentially cancerous) and responsive (differentiated) subpopulations on the basis of their total response profile, observing significant differences in channel capacity. Separating nonresponsive (potentially cancerous) cells from responsive ones may produce purified subpopulations that may respond differently to targeted anticancer therapies in the short-term [22]. Clustering cells into similar response phenotypes also serves to decrease extrinsic noise, but still renders them susceptible to intrinsic noise [18]. Each subpopulation has distinct information transmission capacities. As evidenced by Fig. 7, mixing subpopulations understates the network’s channel capacity.

The concept of mutual information is crucial to understanding the limits by which effective cell signaling can translate to effective cell decision making, given uncertainty in both the intracellular machinery and the extracellular environment. It is often the case that relative differences in concentrations between upstream components of a pathway are discerned by downstream components, not their absolute concentrations [2]. The accuracy of this mapping between external signals and internal states is a clear indicator of signal processing complexity [1]. Mutual information and channel capacity, as constituted in this work, may greatly oversimplify the myriad of informational transactions occurring between and within cells [23]. There may be more complex networks of intracellular relationships beyond a given mathematical model that the mutual information may not account for [2]. Furthermore, experimental noise may confound key measurements of the underlying system. Maximizing signal distributions may be physiologically unrealistic and overly optimistic in comparison to the true distribution [1].

All of the signals considered here could be encoded in exactly 4 bits. The channel capacity values obtained in this study varied between 1.5 and 3 bits. This may be due to inclusion of noise in a theoretical-like analysis. Common signaling motifs were found to contain 4-6 bits of information analytically, whereas the majority of biological systems transmit less than 1 bit experimentally [24, 25]. This discrepancy was speculated to be attributed to the functional necessity of real-world signaling networks and the realization of extrinsic noise in signal transduction in vivo.

The multivariate channel capacity algorithm provided improved estimates of the information transmitted from CT to GFAP in the presence of noise. However, it is not without its drawbacks. The memory capacity of a cell to store vector information over time is a limited resource and can be subject to noise [19, 24]. The information transmitted eventually saturates regardless of which time points are memorized [19]. The k-nearest neighbors density estimation method may misperform for certain response and signal distributions. Extrapolating the channel capacity to an infinite sample size may introduce some bias [3].

Future work will expand on the findings presented in this work. Alternative input stimulation types can be examined for differences in information transmission, like previous studies [11, 18]. The absence of explicit cell-to-cell communication prevents a deeper analysis of the interdependencies of a complex signaling system, wherein cells would influence its nearby neighbors. However, heterogeneity at the single cell level was found to occur through stochastic state transitions between cancer cell phenotypes, not intercellular signaling [22]. Purified cell subpopulations gradually revert to (mixed) equilibrium proportions over time, during which cells transition stochastically between states. Modeling cell state transitions in stochastic signaling networks may be a fruitful avenue of research to elucidating the information content. The multiple signaling pathways that form a network render it robust to information loss due to noise [21]. Isolating the parallel signaling pathways that contribute to glioma differentiation may also shed light on which pathways bear the weight of, and compensate for changes in, information flow [5]. Information is often lost as it traverses through the network, an example of the data processing inequality [3, 5]. Cell-fate processes, such as differentiation, entail a sequence of important intermediate steps where binary decisions take place [1].

## Conclusions

We have proposed an information theoretic framework to examine the information transmission properties of a signaling pathway models related to glioma differentiation. Inhibiting positive feedback mechanisms improved the channel capacity. Increases in information transmission were observed when areas of maximum dynamical variation and similar response dynamics were emphasized. The channel capacity provides a suitable measure of the efficiency of the information transmitted between signal and response components in the glioma differentiation pathway.

## Additional file

Additional file 1 Supplementary Information. Section S1 AN Model Description. Section S2 CLE Model Description. **Figure S1.** AN response dynamics. **Figure S2.** Heatmaps for summary descriptors of AN model. **Figure S3.** CLE- response dynamics. **Figure S4.** Heatmaps for summary descriptors of CLE- model.**Figure S5.** Channel capacities for summary descriptors of AN model as a function of *k* specified in *k*-nearest neighbors algorithm. **Figure S6.** Channel capacities for summary descriptors of CLE model as a function of *k* specified in *k*-nearest neighbors algorithm. **Figure S7.** Channel capacities for summary descriptors of CLE- model as a function of *k* specified in *k*-nearest neighbors algorithm. **Figure S8.** Channel capacities for multivariate response vectors of AN, CLE, and CLE- models as a function of vector dimension *d* specified in channel capacity algorithm. **Table S1.** Initial conditions for model states. **Table S2.** Parameter values for mathematical model. (PDF 548 kb)

## Data Availability

The datasets used and/or analysed during the current study are available at https://github.com/asai2019/glioma-differentiation-sde. The initial conditions of model states are provided in Additional file 1: Table S1. The values of parameters used for model simulations are provided in Additional file 1: Table S2.

## Abbreviations

AN: Additive noise
AUC: Area under the curve
CLE-: Chemical Langevin equation with cyclin D1 inhibition
CLE: Chemical Langevin equation
CT: Cholera toxin
GFAP: Gilial fibrillary acidic protein
HH: High intrinsic noise, high extrinsic noise
HL: High intrinsic noise, low extrinsic noise
LH: Low intrinsic noise, high extrinsic noise
LL: Low intrinsic noise, low extrinsic noise
SDE: Stochastic differential equation
