# Supplementary material for: Exploring the information transmission properties of noise-induced dynamics: application to glioma differentiation
Source: BMC Bioinformatics. 2019 Jul 4;20:375. doi: 10.1186/s12859-019-2970-7 (PMC6610902; doi:10.1186/s12859-019-2970-7)
Supplement: Supplementary file 1 — Supplementary Information. Section S1 AN Model Description. Section S2 CLE Model Description. Figure S1. AN response dynamics. Figure S2. Heatmaps for summary descriptors of AN model. Figure S3. CLE- response dynamics. Figure S4. Heatmaps for summary descriptors of CLE- model.Figure S5. Channel capacities for summary descriptors of AN model as a function of k specified in k-nearest neighbors algorithm. Figure S6. Channel capacities for summary descriptors of CLE model as a function of k specified in k-nearest neighbors algorithm. Figure S7. Channel capacities for summary descriptors of CLE- model as a function of k specified in k-nearest neighbors algorithm. Figure S8. Channel capacities for multivariate response vectors of AN, CLE, and CLE- models as a function of vector dimension d specified in channel capacity algorithm. Table S1. Initial conditions for model states. Table S2. Parameter values for mathematical model. (PDF 548 kb) [file 12859_2019_2970_MOESM1_ESM.pdf]

Supplementary Material for *Exploring the Information Transmission Properties of Noise-induced Dynamics: Application to Glioma Differentiation*

Aditya Sai, Nan Kong

S1 AN Model Description

$$d[PKA] = a_1 dt + \frac{V_1 \cdot CT^{n_1}}{K_1 + CT^{n_1}} dt - d_1[PKA]dt + \sigma_1 dB_1 \quad (1)$$

$$d[CREB] = \frac{V_2 \cdot [PKA]}{K_2 + [PKA]} dt - d_2[CREB]dt + \sigma_2 dB_2 \quad (2)$$

$$d[P13K] = \frac{1}{1 + \frac{[PKA]}{K_3}} dt - d_3[P13K]dt + \sigma_3 dB_3 \quad (3)$$

$$d[AKT] = \frac{V_4 \cdot [P13K]}{K_4 + [P13K]} dt - d_4[AKT]dt + \sigma_4 dB_4 \quad (4)$$

$$d[pGSK3\beta] = \frac{V_5 \cdot ([GSK3\beta_T] - [pGSK3\beta]) \cdot [AKT]}{K_5 + [GSK3\beta_T] - [pGSK3\beta]} dt - d_5[pGSK3\beta]dt + \sigma_5 dB_5 \quad (5)$$

$$d[CyclinD1] = \frac{V_6 \cdot [CyclinD1]^{n_2}}{K_{6a}^{n2} + [CyclinD1]^{n_2}} dt - \frac{d_6 \cdot ([GSK3\beta_T] - [pGSK3\beta]) \cdot [CyclinD1]}{K_{6b} + [GSK3\beta_T] - [pGSK3\beta]} dt + \sigma_6 dB_6 \quad (6)$$

$$d[IL6] = \frac{V_7 \cdot [PKA]}{K_7 + [PKA]} dt - d_7[IL6]dt + \sigma_7 dB_7 \quad (7)$$

$$d[JAK2] = \frac{V_8 \cdot [IL6]}{K_8 + [IL6]} dt - d_8[JAK2]dt + \sigma_8 dB_8 \quad (8)$$

$$d[STAT3] = \frac{V_9 \cdot [JAK2]}{K_9 + [JAK2]} dt - d_9[STAT3]dt + \sigma_9 dB_9 \quad (9)$$

$$d[GFAP] = \Big(\frac{C - [CyclinD1]}{C}\Big)^+ \cdot \frac{V_{10ab} \cdot [CREB]}{K_{10a} + [CREB]} \cdot \frac{[STAT3]}{K_{10b} + [STAT3]} \cdot \frac{V_{10c} \cdot ([GSK3\beta_T] - [pGSK3\beta])^{n_3}}{K_{10c} + ([GSK3\beta_T] - [pGSK3\beta])^{n_3}} dt \quad (10)$$
$$- d_{10}[GFAP]dt + \sigma_{10} dB_{10},$$

where

$$(x)^+ = \begin{cases} x & x > 0 \\ 0 & x \leq 0, \end{cases}$$

and dB_i, $i = 1, \ldots, 10$, are independent, standard Brownian motion processes. We consider values of $\sigma_i = \{0.001, 0.01, 0.05, 0.1\}$, $i = 1, \ldots, 10$.

S2 CLE Model Description

$$\frac{d[PKA]}{dt} = a_1 + \frac{V_1 \cdot CT^{n_1}}{K_1 + CT^{n_1}} - d_1[PKA] + \frac{1}{\sqrt{V}}\left[\sqrt{a_1 + \frac{V_1 \cdot CT^{n_1}}{K_1 + CT^{n_1}}}\zeta_1(t) - \sqrt{d_1[PKA]}\zeta_2(t)\right] \tag{11}$$

$$\frac{d[CREB]}{dt} = \frac{V_2 \cdot [PKA]}{K_2 + [PKA]} - d_2[CREB] + \frac{1}{\sqrt{V}}\left[\sqrt{\frac{V_2 \cdot [PKA]}{K_2 + [PKA]}}\zeta_3(t) - \sqrt{d_2[CREB]}\zeta_4(t)\right] \tag{12}$$

$$\frac{d[P13K]}{dt} = \frac{1}{1 + \frac{[PKA]}{K_3}} - d_3[P13K] + \frac{1}{\sqrt{V}}\left[\sqrt{\frac{1}{1 + \frac{[PKA]}{K_3}}}\zeta_5(t) - \sqrt{d_3[P13K]}\zeta_6(t)\right] \tag{13}$$

$$\frac{d[AKT]}{dt} = \frac{V_4 \cdot [P13K]}{K_4 + [P13K]} - d_4[AKT] + \frac{1}{\sqrt{V}}\left[\sqrt{\frac{V_4 \cdot [P13K]}{K_4 + [P13K]}}\zeta_7(t) - \sqrt{d_4[AKT]}\zeta_8(t)\right] \tag{14}$$

$$\frac{d[pGSK3\beta]}{dt} = \frac{V_5 \cdot ([GSK3\beta_T] - [pGSK3\beta]) \cdot [AKT]}{K_5 + [GSK3\beta_T] - [pGSK3\beta]} - d_5[pGSK3\beta] \tag{15}$$

$$+ \frac{1}{\sqrt{V}}\left[\sqrt{\frac{V_5 \cdot ([GSK3\beta_T] - [pGSK3\beta]) \cdot [AKT]}{K_5 + [GSK3\beta_T] - [pGSK3\beta]}}\zeta_9(t) - \sqrt{d_5[pGSK3\beta]}\zeta_{10}(t)\right]$$

$$\frac{d[CyclinD1]}{dt} = \frac{V_6 \cdot [CyclinD1]^{n_2}}{K_{6a}^{n2} + [CyclinD1]^{n_2}} - \frac{d_6 \cdot ([GSK3\beta_T] - [pGSK3\beta]) \cdot [CyclinD1]}{K_{6b} + [GSK3\beta_T] - [pGSK3\beta]}$$

$$+ \frac{1}{\sqrt{V}}\left[\sqrt{\frac{V_6 \cdot [CyclinD1]^{n_2}}{K_{6a}^{n2} + [CyclinD1]^{n_2}}}\zeta_{11}(t) - \sqrt{\frac{d_6 \cdot ([GSK3\beta_T] - [pGSK3\beta]) \cdot [CyclinD1]}{K_{6b} + [GSK3\beta_T] - [pGSK3\beta]}}\zeta_{12}(t)\right] \tag{16}$$

$$\frac{d[IL6]}{dt} = \frac{V_7 \cdot [PKA]}{K_7 + [PKA]} - d_7[IL6] + \frac{1}{\sqrt{V}}\left[\sqrt{\frac{V_7 \cdot [PKA]}{K_7 + [PKA]}}\zeta_{13}(t) - \sqrt{d_7[IL6]}\zeta_{14}(t)\right] \tag{17}$$

$$\frac{d[JAK2]}{dt} = \frac{V_8 \cdot [IL6]}{K_8 + [IL6]} - d_8[JAK2] + \frac{1}{\sqrt{V}}\left[\sqrt{\frac{V_8 \cdot [IL6]}{K_8 + [IL6]}}\zeta_{15}(t) - \sqrt{d_8[JAK2]}\zeta_{16}(t)\right] \tag{18}$$

$$\frac{d[STAT3]}{dt} = \frac{V_9 \cdot [JAK2]}{K_9 + [JAK2]} - d_9[STAT3] + \frac{1}{\sqrt{V}}\left[\sqrt{\frac{V_9 \cdot [JAK2]}{K_9 + [JAK2]}}\zeta_{17}(t) - \sqrt{d_9[STAT3]}\zeta_{18}(t)\right] \tag{19}$$

$$\frac{d[GFAP]}{dt} = \left(\frac{C - [CyclinD1]}{C}\right)^+ \cdot \frac{V_{10ab} \cdot [CREB]}{K_{10a} + [CREB]} \cdot \frac{[STAT3]}{K_{10b} + [STAT3]} \cdot \frac{V_{10c} \cdot ([GSK3\beta_T] - [pGSK3\beta])^{n_3}}{K_{10c} + ([GSK3\beta_T] - [pGSK3\beta])^{n_3}} \tag{20}$$

$$- d_{10}[GFAP]$$

$$+ \frac{1}{\sqrt{V}}\left[\sqrt{\left(\frac{C - [CyclinD1]}{C}\right)^+ \cdot \frac{V_{10ab} \cdot [CREB]}{K_{10a} + [CREB]} \cdot \frac{[STAT3]}{K_{10b} + [STAT3]}} \right. \tag{21}$$

$$\left. \sqrt{\cdot \frac{V_{10c} \cdot ([GSK3\beta_T] - [pGSK3\beta])^{n_3}}{K_{10c} + ([GSK3\beta_T] - [pGSK3\beta])^{n_3}}}\zeta_{19}(t)\sqrt{d_{10}[GFAP]}\zeta_{20}(t)\right] \tag{22}$$

where V is the total number of molecules for each protein, $\zeta_i(t)$, $i = 1, \ldots, 20$, are temporally uncorrelated, independent Gaussian white noise. Each parameter P_j, $j = 1, \ldots, 39$ is also varied as $P_j(1 + \lambda\epsilon_i)$, where ϵ_i is a standard normal random variable. Therefore, intrinsic noise has a standard deviation of $\frac{1}{\sqrt{V}}$, and extrinsic noise has a standard deviation of λ. We consider values of $\frac{1}{\sqrt{V}} = \{0.001, 0.1\}$ and $\lambda = \{0.001, 0.1\}$. The CLE- model was obtained by multiplying parameter K_{6a} by 10.

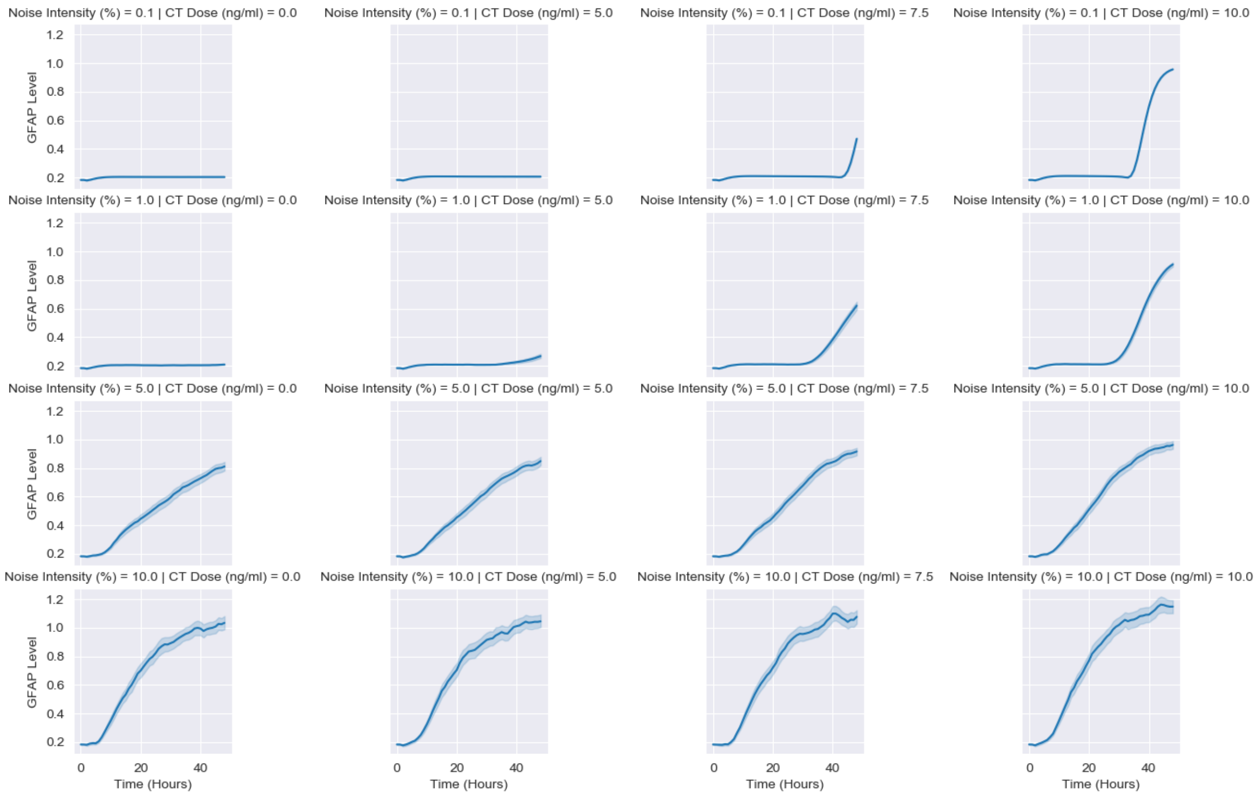

Figure S1: AN response dynamics. Time courses of GFAP level are shown, corresponding to 500 simulated cells exposed to different signals composed of CT dose and noise (expressed as noise intensity). Dark blue lines represent average GFAP level, with shaded areas indicating 95% confidence intervals.

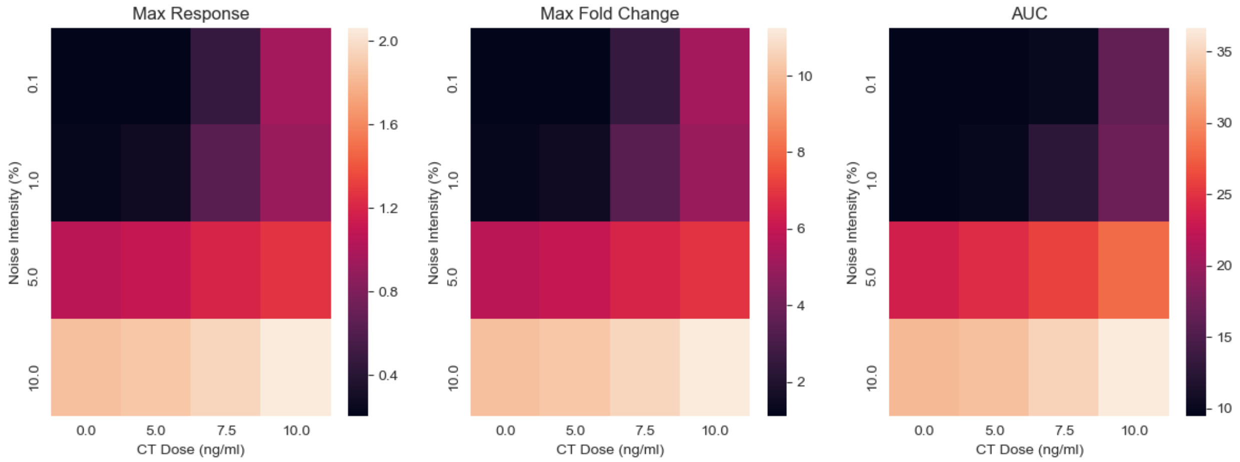

Figure S2: Heatmaps for summary descriptors of AN model. Average maximum response (*left*), maximum fold change (*center*), and area under the curve (*right*) values were calculated for the simulated cell population exposed to each signal.

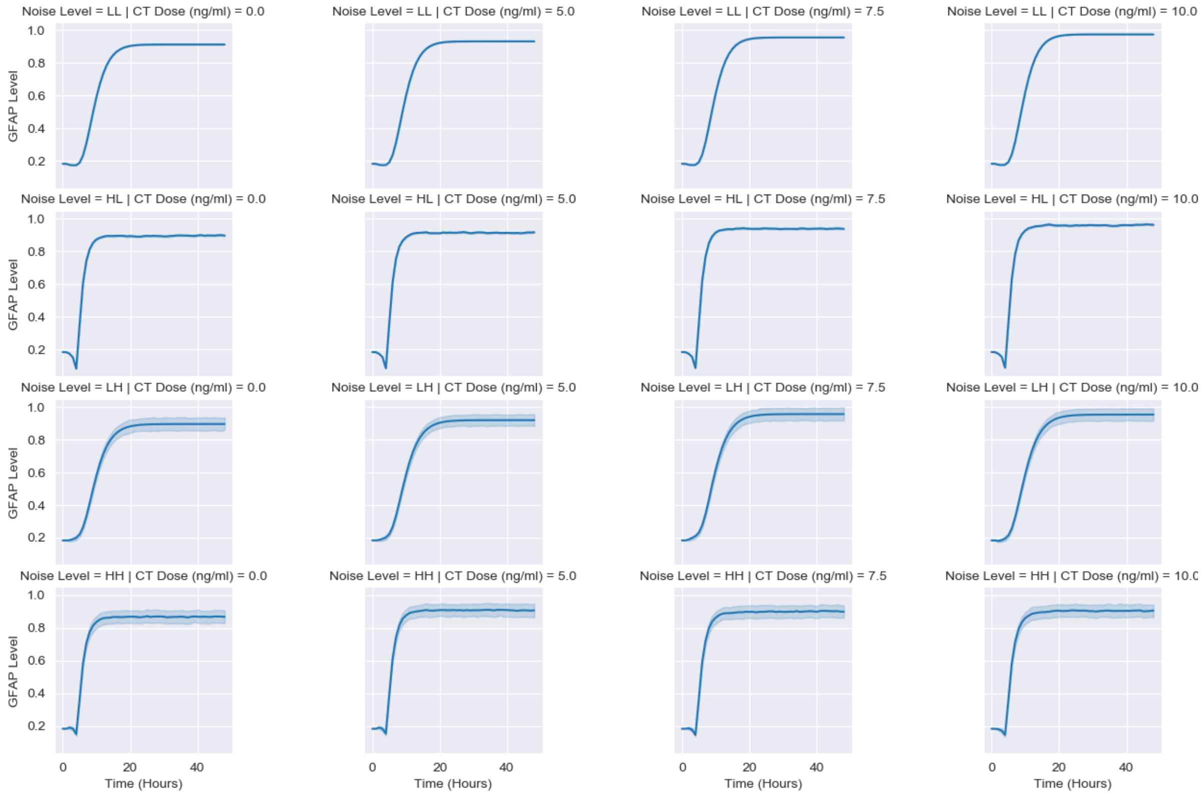

Figure S3: CLE- response dynamics. Time courses of GFAP level are shown, corresponding to 500 simulated cells exposed to different signals composed of CT dose and noise (intrinsic & extrinsic noise). Dark blue lines represent average GFAP level, with shaded areas indicating 95% confidence intervals. Noise levels are defined in Table 1, in main manuscript.

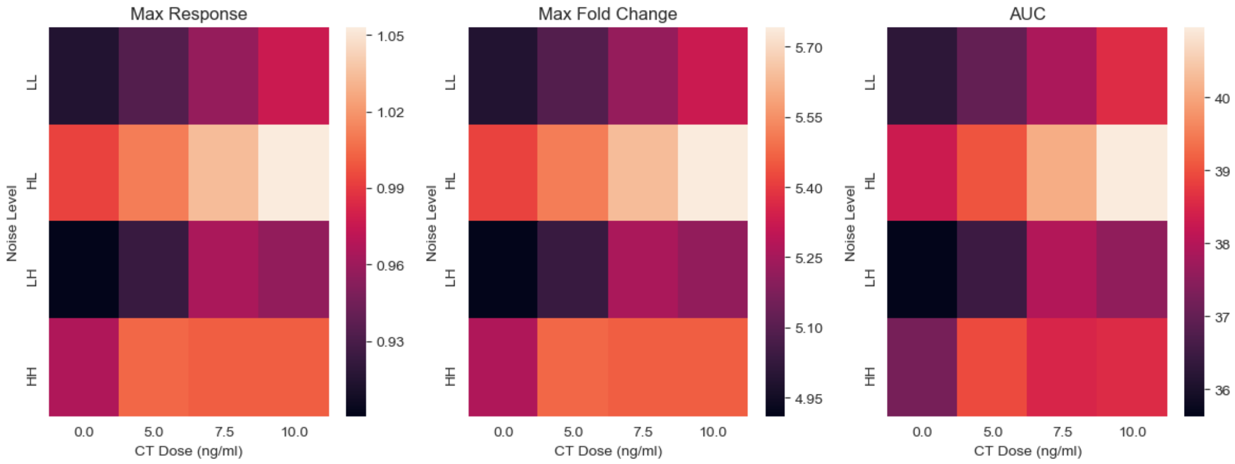

Figure S4: Heatmaps for summary descriptors of CLE- model. Average maximum response (*left*), maximum fold change (*center*), and area under the curve (*right*) values were calculated for the simulated cell population exposed to each signal. Noise levels are defined in Table 1.

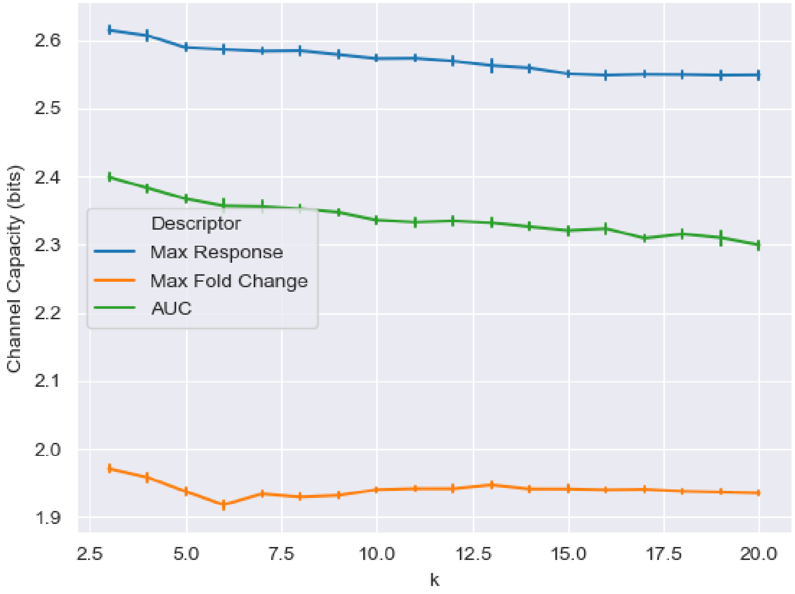

Figure S5: Channel capacities for summary descriptors of AN model as a function of k specified in k-nearest neighbors algorithm.

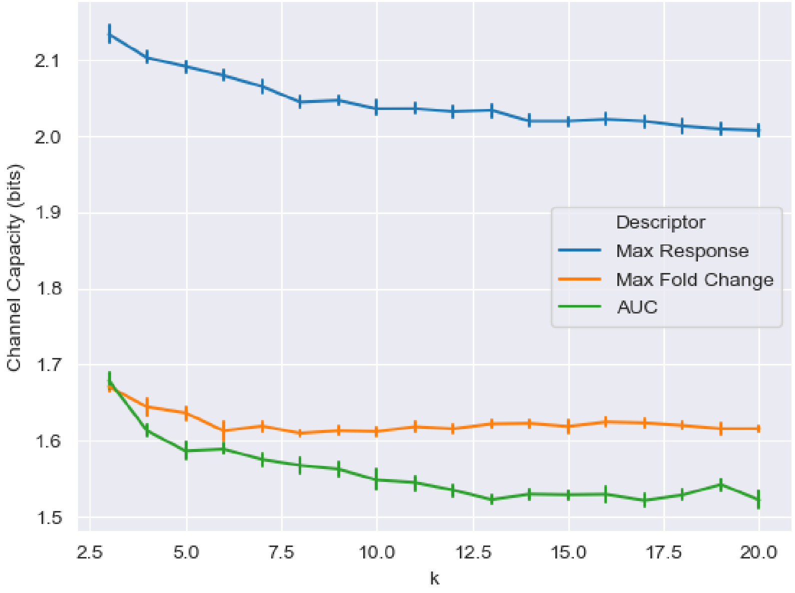

Figure S6: Channel capacities for summary descriptors of CLE model as a function of k specified in k-nearest neighbors algorithm.

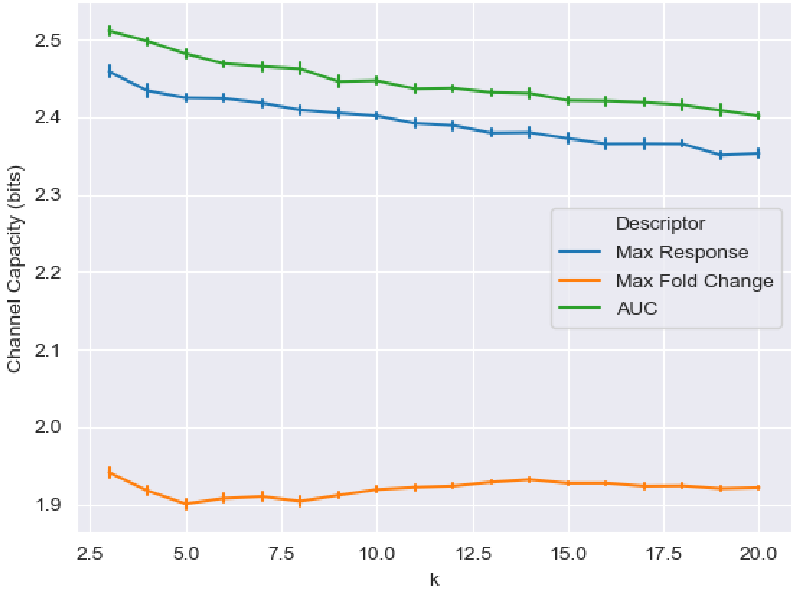

Figure S7: Channel capacities for summary descriptors of CLE- model as a function of k specified in k-nearest neighbors algorithm.

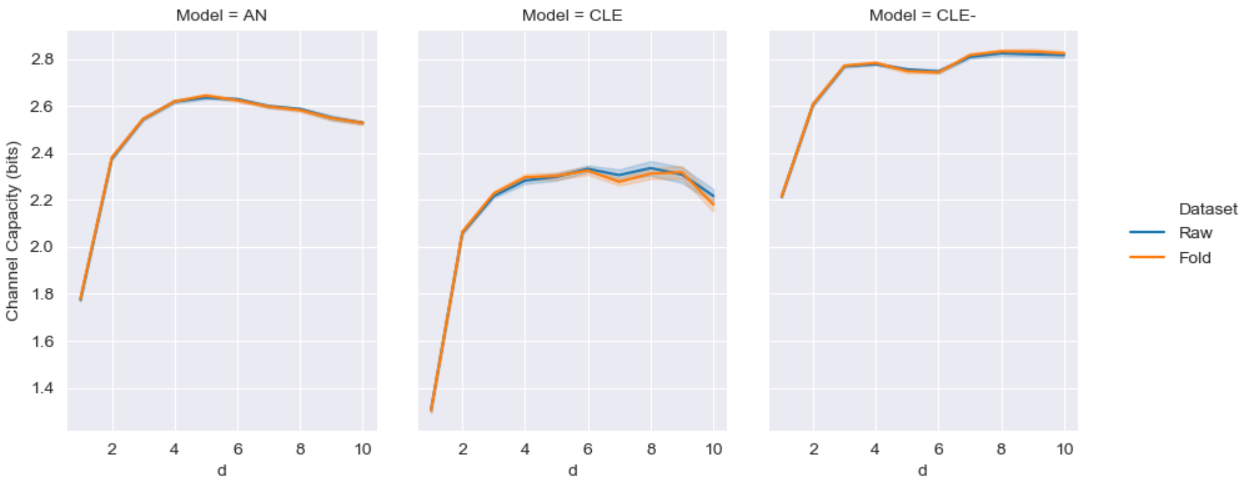

Figure S8: Channel capacities for multivariate response vectors of AN, CLE, and CLE- models as a function of vector dimension d specified in channel capacity algorithm. Results for both original and fold-transformed dynamics are shown.

Table S1: Initial conditions for model states [1, 2].

State. No.	Symbol	Initial Condition
1	$[PKA]$	1.6245
2	$[CREB]$	0.2600
3	$[P13K]$	1.0000
4	$[AKT]$	1.0000
5	$[GSK3\beta]$	0.3000
6	$[CyclinD1]$	1.0500
7	$[IL6]$	0.7239
8	$[JAK2]$	0.4080
9	$[STAT3]$	0.6818
10	$[GFAP]$	0.1834

Table S2: Parameter values for mathematical model [1, 2].

Para. No.	Symbol	Value	Description
1	V_1	1.6245	maximal activation velocity of PKA by Cholera toxin (CT)
2	K_1	9.5398	Michaelis constant of PKA by CT
3	V_2	0.6602	maximal activation velocity of CREB by PKA
4	K_2	0.5351	Michaelis constant of CREB by PKA
5	K_3	0.7018	Michaelis constant of P13K inhibition by PKA
6	V_4	0.1136	maximal activation velocity of AKT by P13K
7	K_4	0.4037	Michaelis constant of AKT by P13K
8	V_5	0.4449	maximal phosphorylation velocity of GSK3β by AKT
9	K_5	0.2951	Michaelis constant of GSK3β by AKT
10	V_6	0.5448	maximal self-amplification velocity of cyclin D1
11	K_{6a}	0.9413	Michaelis constant of cyclin D1
12	K_{6b}	0.8489	Michaelis constant of cyclin D1 vt GSK3β
13	V_7	0.6390	maximal activation velocity of IL6 by PKA
14	K_7	0.2324	Michaelis constant of IL6 inhibition by PKA
15	V_8	0.5359	maximal activation velocity of IL6 by PKA
16	K_8	0.2558	Michaelis constant of JAK2 by IL6
17	V_9	0.7861	maximal activation velocity of STAT3 by JAK2
18	K_9	0.6108	Michaelis constant of STAT3 by JAK2
19	V_{10ab}	0.0841	maximal activation velocity of GFAP by CREB
20	K_{10a}	0.1316	Michaelis constant of GFAP by CREB
21	K_{10b}	0.1692	Michaelis constant of GFAP by STAT3
22	V_{10c}	0.5249	maximal activation velocity of GFAP by GSK3β
23	K_{10c}	0.7371	Michaelis constant of GFAP by GSK3β
24	K_{10d}	0.8384	Michaelis constant of GFAP by cyclin D1
25	d_1	0.4668	dephosphorylation rate of PKA
26	d_2	0.5774	dephosphorylation rate of CREB
27	d_3	0.4602	dephosphorylation rate of P13K
28	d_4	0.7700	dephosphorylation rate of AKT
29	d_5	0.7147	dephosphorylation rate of pGSK3β
30	d_6	0.8363	dephosphorylation rate of cyclin D1
31	d_7	0.7163	dephosphorylation rate of IL6
32	d_8	0.9704	dephosphorylation rate of JAK2
33	d_9	0.7398	dephosphorylation rate of STAT3
34	d_{10}	0.4881	dephosphorylation rate of GFAP
35	C	1.25	maximal steady state of cyclin D1
36	CT	10	CT dose (ng/ml)
37	n_1	3	Hill coefficient of PKA activation induced by CT
38	n_2	10	Hill coefficient of cyclin D1 feedback
39	n_3	8	Hill coefficient of GFAP activation promoted by active GSK3β
40	$GSK3\beta_T$	1	total amount of GSK3β
41	a_1	0.4668	base activation rate of PKA due to cAMP

References

[1] Sun, X., Zheng, X., Zhang, J., Zhou, T., Yan, G., Zhu, W.: Mathematical modeling reveals a critical role for cyclin D1 dynamics in phenotype switching during glioma differentiation. FEBS Letters **589**(18), 2304–2311 (2015)

[2] Sun, X., Zhang, J., Zhao, Q., Chen, X., Zhu, W., Yan, G., Zhou, T.: Stochastic modeling suggests that noise reduces differentiation efficiency by inducing a heterogeneous drug response in glioma differentiation therapy. BMC Systems Biology **10**(1), 73 (2016)
